# Cost-Effectiveness of Cranberry Capsules to Prevent Urinary Tract Infection in Long-Term Care Facilities: Economic Evaluation with a Randomized Controlled Trial

**DOI:** 10.1111/jgs.12595

**Published:** 2014-01-17

**Authors:** Wilbert B van den Hout, Monique A A Caljouw, Hein Putter, Herman J M Cools, Jacobijn Gussekloo

**Affiliations:** * Department of Medical Decision Making, Leiden University Medical CenterLeiden, the Netherlands; † Department of Public Health and Primary Care, Leiden University Medical CenterLeiden, the Netherlands; ‡ Department of Medical Statistics, Leiden University Medical CenterLeiden, the Netherlands

**Keywords:** economic evaluation, geriatrics, long-term care facility, urinary tract infection, prevention, cranberry

## Abstract

**Objectives:**

To investigate whether the preventive use of cranberry capsules in long-term care facility (LTCF) residents is cost-effective depending on urinary tract infection (UTI) risk.

**Design:**

Economic evaluation with a randomized controlled trial.

**Setting:**

Long-term care facilities.

**Participants:**

LTCF residents (N = 928, 703 female, median age 84), stratified according to UTI risk.

**Measurements:**

UTI incidence (clinically or strictly defined), survival, quality of life, quality-adjusted life years (QALYs), and costs.

**Results:**

In the weeks after a clinical UTI, participants showed a significant but moderate deterioration in quality of life, survival, care dependency, and costs. In high-UTI-risk participants, cranberry costs were estimated at €439 per year (1.00 euro = 1.37 U.S. dollar), which is €3,800 per prevented clinically defined UTI (95% confidence interval = €1,300–infinity). Using the strict UTI definition, the use of cranberry increased costs without preventing UTIs. Taking cranberry capsules had a 22% probability of being cost-effective compared with placebo (at a willingness to pay of €40,000 per QALY). In low-UTI-risk participants, use of cranberry capsules was only 3% likely to be cost-effective.

**Conclusion:**

In high-UTI-risk residents, taking cranberry capsules may be effective in preventing UTIs but is not likely to be cost-effective in the investigated dosage, frequency, and setting. In low-UTI-risk LTCF residents, taking cranberry capsules twice daily is neither effective nor cost-effective.

Urinary tract infection (UTI) is a common bacterial infection in residents of long-term care facilities (LTCFs).^1^–^4^ The effectiveness of the use of cranberry capsules to prevent UTIs was assessed in a randomized controlled trial.^5^ In residents with high UTI risk, taking cranberry capsules twice daily reduced the incidence of clinically defined UTI by 26%. No reduction was found for strictly defined UTI or in residents with low UTI risk. The current brief report investigates the effect of UTI on health and costs and whether the preventive use of cranberry capsules in LTCFs is cost-effective.

## Methods

This economic evaluation was part of a double-blind randomized placebo-controlled multicenter trial.^5^ Residents from LTCFs (N = 928, median age 84, 703 female) were randomized to receive cranberry or placebo capsules twice daily for 12 months. The cranberry capsules contained 500 mg of the product with 1.8% proanthocyanidins (9 mg). Participants were stratified according to UTI risk (including long-term catheterization, diabetes mellitus, ≥1 UTIs in the preceding year). Main outcomes of the trial were incidence of UTI according to a clinical definition (following clinical practice guidelines for residents in LTCFs) and a strict definition (with confirmation by a positive dipslide or culture).^5^

### Cost-Effectiveness and Cost-Utility Analysis

The economic evaluation consisted of a cost-effectiveness analysis (CEA) from a narrow perspective and a cost-utility analysis (CUA) from a lifelong societal perspective for high- and for low-UTI-risk participants.

The CEA from a narrow perspective was based directly on the trial data during follow-up, to prevent modeling assumptions. Effectiveness was measured according to the number of clinically defined and strictly defined UTIs (first and recurrent). Costs included only cranberry use.

The CUA was performed from a lifelong societal perspective. Costs, survival and quality-adjusted life years (QALYs) were estimated using a non-Markovian state-transition model, with parameters estimated from the trial data. In this model, the cranberry and placebo groups differed in their clinically defined UTI infection rate but not in the consequences per UTI. Thus, it was implicitly assumed that prevented UTIs are comparable with nonprevented UTIs and that cranberry use has no relevant effects other than cranberry costs and UTI prevention. These modeling assumptions were made beforehand because it was clear that the study would have insufficient power for a direct randomized economic comparison.

**Figure 1:**
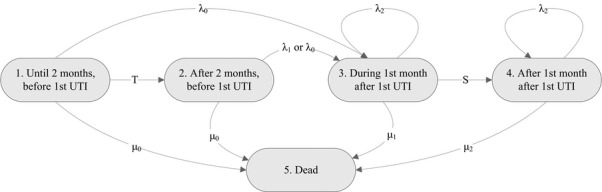
The state-transition model used in the economic evaluation. UTI = Urinary Tract Infection.

In the CUA model (Figure1), participant time was categorized into five model states: during the initial 2 months and before the first UTI, after the initial 2 months and before the first UTI, during the first month after the first UTI, after the first month after the first UTI, and death.

### UTI Infection Rate and Mortality

Three separate annual UTI infection rates were estimated in a combined Poisson regression analysis. Infection rates in the cranberry and placebo groups were different after the first 2 months and before the first UTI (State 2). No effect of cranberry use was seen in the initial 2 months (State 1) or after the first UTI (States 3 and 4). Occurrence of a first UTI was associated with greater mortality during the subsequent month (State 3) and after the subsequent month (State 4). Annual mortality was estimated in a combined Poisson regression analysis.

### Utilities

Utilities represent the valuation of the quality of life of the participants on a scale anchored at 1 (perfect health) and 0 (as bad as being dead). Utility was measured using the EQ-5D classification system, which is a brief questionnaire with five domains (mobility, self-care, usual activities, pain and discomfort, and anxiety and depression), each with three levels (no, some, or extreme problems).^6^ Utility values were assigned to the EQ-5D using the Dutch tariff.^7^ Valuations were also obtained using a visual analog scale (VAS) ranging from 100 (perfect health) to 0 (worst imaginable health), which was transformed to a utility scale using a power transformation.^8^ EQ-5D and VAS measurements were obtained from the participants (11%) or from well-informed nurses or caregivers (89%).

Utility before and after the first UTI (parameters *U*_0_ and *U*_2_) was estimated from EQ-5D and VAS measurements obtained at baseline and after 6 and 12 months (correcting for time). In addition, UTIs were assumed to have a short-term effect on utility for 2 weeks. Additional EQ-5D and VAS measurements were obtained in 123 participants with a clinically defined UTI (from 17 different LTCFs) every 3 days over 3 weeks after the UTI to estimate this effect. The utility decrement during the UTI was estimated as the difference between the average over the first 2 weeks and the average over the third week (parameter Δ*U,* attributed to State 3 with parameter *U*_1_ = *U*_2_−Δ*U* × 14/30).

### Costs

The economic model included two types of costs. The first was the costs of cranberry use (parameter *c*_1_). These costs were estimated at €439 annually (€0.62 per intake) based on one capsule twice a day, a market price of €44 for 180 capsules, on average 45 seconds of nursing time per capsule (estimated using time registrations), nursing time valued at €30 per hour,^9^ and 97% adherence (1.00 euro = 1.37 U.S. dollar).

The second type of costs were the costs associated with each UTI (parameter *c*_2_, Table1), including costs of UTI diagnostics and antibiotic treatment, additional care by the elderly-care physician, additional nursing care, and hospitalizations. Costs of UTI diagnostics and antibiotic treatment per UTI were calculated from actual costs in patient records for each UTI (n = 548). Additional care by the elderly-care physician was estimated at on average €25 per UTI (10–30 minutes of time, valued at €111 per hour^9^,^10^). Additional nursing costs during the 2 weeks after a UTI were estimated in proportion to the Care Dependency Scale,^11^ which measures 15 items of basic care needs, each rated on a 5-point scale (1 = completely dependent; 5 = completely independent). Hospitalizations costs were recorded for six UTIs (1% of n = 548), all in high-UTI-risk participants. Costs per hospitalization ranged from €3,000 (for 6 days of normal hospital care) to €15,000 (for 7 days of normal care and 5 days of intensive care).^9^

**Table 1:** Parameters for the Health–Economic Model, Estimated from the Trial Data

Parameter	Estimated Value	(95% Confidence Interval)
UTI infection rates^b^ in low-UTI-risk participants		
Rate before the first UTI, during the first 2 months (*λ*_0_)	0.32	(0.23–0.40)
Hazard ratio before first UTI,after first 2 months (*λ*_1_/*λ*_0_)^a^,^c^	1.41	(0.77–1.86)
Hazard ratio after the first UTI (*λ*_2_/*λ*_0_)^c^	4.02	(2.11–5.29)
UTI infection rates^b^ in high-UTI-risk participants		
Rate before the first UTI, during the first 2 months (*λ*_0_)	0.81	(0.68–0.94)
Hazard ratio before first UTI, after first 2 months (*λ*_1_/*λ*_0_)^a^,^c^	0.75	(0.52–0.94)
Hazard ratio after the first UTI (*λ*_2_/*λ*_0_)^c^	1.81	(1.35–2.18)
Mortality^b^		
Rate before the first UTI (*μ*_0_)	0.33	(0.29–0.38)
Hazard ratio during the first month after first UTI (*μ*_1_/*μ*_0_)^c^	3.57	(2.00–4.81)
Hazard ratio after the first month after first UTI (*μ*_2_/*μ*_0_)^c^	1.32	(0.87–1.67)
Utilities^d^ based on EQ-5D		
Before the first UTI (U_0_)	0.37	(0.36–0.38)
Decrement after the first UTI (U_2_–U_0_)^c^	0.02	(−0.01–0.05)
Decrement during first 2 weeks after first UTI (ΔU)^c^	0.04	(0.01–0.07)
Utilities^d^ based on visual analog scale		
Before the first UTI (U_0_)	0.73	(0.72–0.74)
Decrement after the first UTI (U_2_–U_0_)	0.00	(−0.02–0.02)
Decrement during first 2 weeks after first UTI (ΔU)	0.03	(0.00–0.05)
Annual costs of cranberry use, € (*c*_1_)^c^	439	—
Cost per UTI, €		
Cost of diagnostics	8	(6–10)
Cost of antibiotic treatment	3	(2–4)
Cost of elderly care physician	25	(22–28)
Cost of additional nursing care	120	(49–194)
Cost of hospitalizations	40	(−8–74)
Total cost per (prevented or nonprevented) UTI (*c*_2_)	196	(111–278)

a Only in the cranberry group.

b Annual event rate.

c Relative or absolute change during the specified period, compared with the base value.

d Valuation of quality of life of the participants on a scale anchored at 1 (perfect health) and 0 (as bad as being dead).

UTI = urinary tract infection.

Costs were presented in euros, at 2013 prices (updated if necessary using the general Dutch consumer price index).^12^ Included costs were all medical costs, which for this trial population coincided with the societal perspective.

### Lifelong Outcomes

The model shown in Figure1 and Table1 was used to extrapolate the trial period to lifelong outcomes. Life expectancy was calculated as the expected total time spent in States 1 to 4. QALYs were calculated by weighing the time in each state with the appropriate utility value, discounted at *δ* = 4%.^13^ Using this approach, the following formula for the difference in discounted QALYs between the cranberry and the placebo groups was derived:

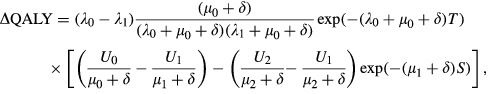
where *T* denotes the initial 2 months and *S* denotes the 1 month after the first UTI. A similar formula was derived for the discounted costs. The models for the cranberry and placebo groups differ only in their value for the infection rate (*λ*_1_ and *λ*_0_, respectively) and in their value for the annual cranberry costs (*c*_1_ and 0, respectively).

### Statistical Analysis

Uncertainty due to sampling error for the estimated lifelong outcomes was assessed using bootstrap analysis (using *B* = 10,000 bootstrap samples). For each bootstrap sample, all model parameters (Table1) were re-estimated, and the lifelong formulae were used to estimate outcome. The 95% confidence intervals (CIs) for the parameters and outcomes were assessed from the 2.5 and 97.5 percentiles among the bootstrap samples.^10^ Statistical analyses for the economic evaluation were performed in R version 2.13.0 (Vienna, Austria).

Depending on the willingness to pay (WTP) for obtained effectiveness, cranberry use is estimated to be cost-effective if it has a better net benefit (NB = WTP × effectiveness−costs) than placebo. Cost-effectiveness acceptability curves were used to plot the probability that cranberry use is more cost-effective than placebo as a function of WTP (estimated as the percentage of bootstrap samples in which cranberry use had a better estimated NB). Confidence intervals for the cost-effectiveness ratio were calculated as WTP values for which the difference in net benefit was not significantly different.^14^ The base-case CUA compared total societal costs with QALYs calculated from the Dutch tariff for the EQ-5D at a WTP of €40,000 per QALY.

## Results

### Effect of UTIs on Mortality, Utility, and Costs

Quality of life was significantly but moderately worse during a UTI; comparing the first 2 weeks after a UTI with the third week, averages were 0.341 versus 0.379 for the EQ-5D (difference 0.038, *P* = .02) and 0.727 versus 0.753 for the VAS (difference 0.026, *P* = .03).

Mortality in the month after a first UTI was 3.6 times as great as in residents without a UTI (Table1). After more than a month, the difference was not statistically significant.

The Care Dependency Scale was also significantly but moderately worse during a UTI (40.7 vs 42.0; difference 1.2; *P* = .01), with an estimated 4% increase in nursing costs in the 2 weeks after a UTI. Total healthcare costs associated with (prevented) UTIs were estimated at €196, primarily consisting of the additional nursing care (61%), followed by hospitalization costs (20%), care by the elderly-care physician (13%), diagnostics (4%). and antibiotic treatment (2%).

### High-UTI-Risk Participants

Cranberry use on average prevented 0.09 clinically defined UTIs (0.69 vs 0.78, *P* = .32) during the trial follow-up (of 289 vs 289 days, *P* = .99). The associated costs were estimated at €3,800 per prevented UTI (95% CI = €1,300–infinity). Cranberry use did not prevent strictly defined UTIs during follow-up (0.28 vs 0.22, *P* = .30).

**Figure 2:**
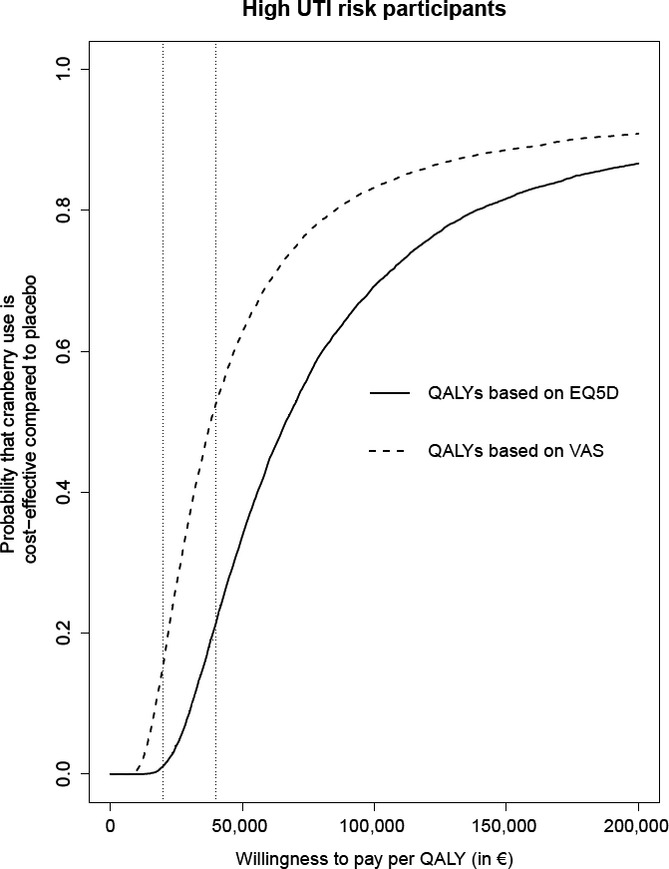
Cost-effectiveness acceptability curves, for high-urinary tract infection (UTI)-risk participants (the probability that cranberry use is more cost-effective than placebo, depending on how much one is willing to pay for a quality-adjusted life year (QALY), based on utility measured using the EQ-5D or the visual analog scale (VAS)).

From a lifetime societal perspective, the reduced clinical UTI infection rate resulted in improvements in other health outcomes and costs, although not significantly (Table2). Life expectancy was estimated to be approximately 2 weeks longer (0.044 years, 95% CI = −0.023–0.091). The savings on costs associated with UTIs were much smaller than the cranberry costs, use of cranberry capsules increased lifelong total costs by €941 (95% CI = €779–1,055). Whether this cost difference is economically acceptable depends on how much one is willing to pay for the health improvement in terms of QALYs (Figure2). For relatively low willingness to pay up to €20,000 per QALY, the probability that cranberry use is more cost-effective than placebo was estimated at less than 1% (in the base-case analysis using the EQ-5D). At €40,000 per QALY, the probability that cranberry use is cost-effective was estimated at 22%. When the VAS was used instead of the EQ-5D, more value was assigned to quality of life during the added life expectancy. As a result, the estimated probability that cranberry use is more cost-effective than placebo at a willingness to pay of €20,000 and €40,000 per QALY was 16% and 53%, respectively (Figure2).

**Table 2:** Mean Lifelong Health-Economic Outcomes of Treatment with or without Cranberry, Estimated from the Health-Economic Model

Outcome	Cranberry	Placebo	Difference	(95% Confidence Interval)
Low UTI risk				
Number of UTIs^a^,^b^	2.13	1.84	0.29	(−0.06–0.60)
Life expectancy, years^a^	2.53	2.59	−0.06	(−0.14–0.05)
QALYs based on EQ-5D^c^	0.83	0.85	−0.02	(−0.05–0.01)
QALYs based on VAS^c^	1.69	1.73	−0.04	(−0.09–0.03)
Cost of cranberry use, €	1,012	0	1,012	(863–1,120)
Cost of diagnostics, €	15	13	2	(−1–5)
Cost of antibiotic treatment, €	5	4	1	(−1–2)
Cost of elderly-care physician, €	47	40	7	(−2–15)
Cost of additional nursing care, €	228	196	32	(−22–68)
Cost of hospitalizations, €	76	65	11	(−13–23)
Total UTI cost, €	1,383	318	1,065	(889–1,183)
High UTI risk				
Number of UTI^a^,^b^	2.75	2.96	−0.21	(−0.42–0.04)
Life expectancy, years^a^	2.45	2.40	0.05	(−0.02–0.09)
QALYs based on EQ-5D^c^	0.81	0.79	0.02	(−0.01–0.03)
QALYs based on VAS^c^	1.64	1.61	0.03	(−0.01–0.06)
Cost of cranberry use, €	982	0	982	(814–1,099)
Cost of diagnostics, €	20	22	−2	(−4–1)
Cost of antibiotic treatment, €	7	8	−1	(−2–1)
Cost of elderly-care physician, €	61	66	−5	(−10–1)
Cost of additional nursing care, €	298	323	−25	(−49–13)
Cost of hospitalizations, €	99	107	−8	(−17–9)
Total UTI cost, €	1,467	526	941	(779–1,055)

a Undiscounted.

b Lifelong, both first and other urinary tract infections (UTIs), using the clinical definition.

c Quality-adjusted life years (QALYs; life expectancy weighed by utility for quality of life).

VAS = Visual Analog Scale.

The economic assessment would be more favorable to cranberry use if the costs of cranberry use were lower or the savings per prevented UTI were higher. The costs of cranberry use would need to decrease from €439 to €300 to make cranberry and placebo equally cost-effective. Similarly, the savings per prevented clinical UTI would need to increase from €196 to €1,704 to make cranberry and placebo equally cost-effective.

### Low-UTI-Risk Participants

In the low-UTI-risk participants, because no effect of cranberry use on UTI infection rate was found, there was also no difference in the other health outcomes. The only difference was the estimated lifelong costs of €1,012 for cranberry use. As a result, it is highly unlikely that use of cranberry capsules is cost-effective in low-UTI-risk residents (probability <3%, regardless of willingness to pay per QALY).

## Discussion

This study investigated whether the use of cranberry capsules is more cost-effective than placebo based on data from a randomized controlled trial.^5^ In participants with low UTI risk, the use of cranberry capsules did not prevent UTIs, and consequently, their use is not cost-effective. In high-UTI-risk participants, there were fewer clinically defined UTIs. Moreover, there was a significant, but moderate, short-term effect of those UTIs on quality of life and care dependency. Most of the QALY gain was due to the prevented UTI mortality, resulting in a gain in life expectancy of approximately 2 weeks. This relative 1.5% improvement in life expectancy is consistent with the estimated mortality attributable to UTI (7.7%) combined with the 26% treatment effect.^5^

Savings on prevented UTIs partly compensate for the costs of the cranberry capsules, but using a lifelong perspective, those savings added up to approximately €50. As a result, the overall cost difference is about equal to the costs of the cranberry capsules, estimated at €439 per year or at €3,800 per prevented clinically defined UTI. The health gain in terms of QALYs was small in comparison with the costs, so use of cranberry capsules was not likely to be cost-effective (22% for a WTP threshold of €40,000 per QALY).

### Options to Improve Cost-Effectiveness

Cranberry use does not constitute a low-cost strategy to prevent UTIs.^15^ The estimated price for the capsules was lower than the price estimated previously (€0.24 vs CAN$0.73 ≈ €0.61),^16^ but the overall costs per capsule were somewhat similar because of the added nursing time in the current study. The previous study estimated the costs per prevented UTI at CAN$1,890, which was acknowledged as quite high.^16^ That study suggested that cranberry use could be cost-effective if the strength or size of the cranberry product could be reduced without reducing effectiveness. In the current study, a preventive effect was seen with the cranberry capsules after 2 months. It is unknown whether lowering the frequency after those 2 months would change not only the costs, but also the effect.

Second, the cost-effectiveness of cranberry use would also be more favorable in settings in which the savings per prevented UTI were higher. In the current study, these savings would need to be eight times as high to make cranberry and placebo equally cost-effective. This seems unrealistic for the Dutch LTCF setting, although in other healthcare systems, residents with UTIs may more frequently be referred to a hospital than the 1% in the current study population. Similarly, in noninstitutionalized vulnerable older persons, the savings associated with prevented UTIs may be higher because of the need for additional formal and informal care during and after a UTI.

Third, the high-UTI-risk criteria included diabetes mellitus, long-term catheterization, and UTI in the preceding year. Better identification of older persons at high UTI risk may also improve cost-effectiveness.

### Limitations

There are several complicating factors in analyzing cost-effectiveness in vulnerable older persons in LTCFs. The first is how to value the residents’ health. According to the EQ-5D, health during the 2-week life expectancy gain was valued at approximately 40%, whereas according to the VAS, this gain would be valued at approximately 70%. In accordance with the study protocol, the EQ-5D was considered to be more appropriate than the VAS because the EQ-5D provides a societal valuation and because the descriptions it requires are less subjective than the valuations that the VAS requires.^17^ Consistent with the high percentage of participants with dementia (76%) and the high percentage of participants for whom family provided informed consent (84%),^5^ nurses mostly provided the utility measures (89%), and little is known about the validity of proxy VAS valuations in a LTCF context. Considering the often poor health of LTCF residents, the VAS valuations appear high.

Another factor is the high costs of standard LTCF care, amounting to approximately €80,000 annually in the Netherlands.^9^,^18^ Including these high costs in the analysis would make any life-prolonging treatment too expensive, even if the treatment itself was cost free. In the analysis, the 2-week life expectancy gain would add approximately €1,500 to the costs associated with cranberry use, confirming the conclusion that use of cranberry capsules is not likely to be cost-effective. Dutch guidelines for economic evaluations in health care recommend including costs associated with additional life time only if they are related to the primary intervention, which was not the case in the current analysis.

Third, it was decided beforehand that the economic state/transition model would be based on the clinical definition, because it was expected that it would be more predictive of outcome. Had the strict UTI definition been used for the model, the estimated effectiveness and cost-effectiveness would have been less favorable to the use of cranberry capsules, confirming the conclusion that use of cranberry capsules is not likely to be cost-effective.

Finally, this study was performed in Dutch LTCFs, where elderly-care physicians provide medical care.^19^–^21^ The results are not automatically generalizable to vulnerable older persons living at home. In LTCFs, medication prescription and distribution are well organized. Because the study capsules were added to the existing drug-dispensing system, participants rarely missed taking a capsule, reflected in a high adherence rate. For other settings, not only differences in vulnerability and infection rates are expected, but also in adherence. Moreover, at home, cranberry capsules can be taken without the help of nurses, which would halve the costs of cranberry use.

## Conclusions

In high-UTI-risk residents, taking cranberry capsules may be effective in preventing UTIs but is not likely to be cost-effective in the investigated dosage, frequency, and setting. In low-UTI-risk LTCF residents, taking cranberry capsules twice daily is neither effective nor cost-effective.
